# The whole transcriptome effects of the PPARα agonist fenofibrate on livers of hepatocyte humanized mice

**DOI:** 10.1186/s12864-018-4834-3

**Published:** 2018-06-07

**Authors:** Montserrat A. de la Rosa Rodriguez, Go Sugahara, Guido J. E. J. Hooiveld, Yuji Ishida, Chise Tateno, Sander Kersten

**Affiliations:** 1 Nutrition, Metabolism and Genomics group, Division of Human Nutrition and Health, Wageningen University, Stippeneng 4, 6708 WE Wageningen, The Netherlands; 2 Research and Development Department, PhoenixBio, Co., Ltd, 3-4-1 Kagamiyama, Higashi-, Hiroshima, Japan; 3 Liver Research Project Center, Hiroshima University, 1-2-3, Kasumi, Minami-ku, Hiroshima, Japan

**Keywords:** Hepatocyte humanized mice, Fenofibrate, PPARα, Transcriptomics, Lipid metabolism, DNA synthesis

## Abstract

**Background:**

The role of PPARα in gene regulation in mouse liver is well characterized. However, less is known about the role of PPARα in human liver. The aim of the present study was to better characterize the impact of PPARα activation on gene regulation in human liver. To that end, chimeric mice containing hepatocyte humanized livers were given an oral dose of 300 mg/kg fenofibrate daily for 4 days. Livers were collected and analyzed by hematoxilin and eosin staining, qPCR, and transcriptomics. Transcriptomics data were compared with existing datasets on PPARα activation in normal mouse liver, human primary hepatocytes, and human precision cut liver slices.

**Results:**

Of the different human liver models, the gene expression profile of hepatocyte humanized livers most closely resembled actual human liver. In the hepatocyte humanized mouse livers, the human hepatocytes exhibited excessive lipid accumulation. Fenofibrate increased the size of the mouse but not human hepatocytes, and tended to reduce steatosis in the human hepatocytes. Quantitative PCR indicated that induction of PPARα targets by fenofibrate was less pronounced in the human hepatocytes than in the residual mouse hepatocytes. Transcriptomics analysis indicated that, after filtering, a total of 282 genes was significantly different between fenofibrate- and control-treated mice (*P* < 0.01). 123 genes were significantly lower and 159 genes significantly higher in the fenofibrate-treated mice, including many established PPARα targets such as *FABP1*, *HADHB*, *HADHA*, *VNN1*, *PLIN2*, *ACADVL* and *HMGCS2*. According to gene set enrichment analysis, fenofibrate upregulated interferon/cytokine signaling-related pathways in hepatocyte humanized liver, but downregulated these pathways in normal mouse liver. Also, fenofibrate downregulated pathways related to DNA synthesis in hepatocyte humanized liver but not in normal mouse liver.

**Conclusion:**

The results support the major role of PPARα in regulating hepatic lipid metabolism, and underscore the more modest effect of PPARα activation on gene regulation in human liver compared to mouse liver. The data suggest that PPARα may have a suppressive effect on DNA synthesis in human liver, and a stimulatory effect on interferon/cytokine signalling.

## Background

The Peroxisome Proliferator Activated Receptors (PPARs) are a group of nuclear receptors involved in the transcriptional regulation of a variety of biological processes, including lipid metabolism and inflammation [1–3]. PPARs regulate gene expression by acting as ligand-activated transcription factors. PPARs interact with DNA as part of a heterodimeric complex with the retinoid X receptor RXR [4–6]. The ligands for PPARs cover a broad range of synthetic and endogenous compounds ranging from environmental contaminants to specific drug classes, fatty acids, eicosanoids, and other lipid species [7]. Three different PPAR subtypes exist in mammals: PPARα, PPARβ/δ, and PPARγ, each with a distinct tissue expression profile and set of functions.

PPARα is expressed in several tissues, particularly in liver, kidney, heart, skeletal muscle and intestine [8, 9]. Studies in mice using whole-body or liver-specific PPARα−/− mice have shown that PPARα is the master regulator of lipid metabolism in the liver during fasting [10–12]. Specifically, fasted PPARα−/− mice suffer from a host of metabolic abnormalities including hypoglycemia, hypoketonemia, elevated plasma non-esterified fatty acids, and a fatty liver. These metabolic defects are rooted in defective transcription of hundreds of genes involved in numerous metabolic pathways covering nearly every aspect of hepatic lipid metabolism [13].

Besides its role as key transcriptional regulator of lipid metabolism during fasting, PPARα is mainly known as the receptor for a diverse group of compounds known as peroxisome proliferators [14, 15]. The group of peroxisome proliferators include plasticizers, insecticides, herbicides, surfactants, organic solvents, and hypolipidemic fibrate drugs [16]. Safety concerns have been raised about these compounds due to their ability to promote hepatocarcinogenesis and the proliferation of peroxisomes in rodent species [17, 18]. Studies using human liver model systems have largely allayed these concerns by failing to find supportive evidence for a proliferative and pro-carcinogenic effect of PPARα ligands in human cells [19].

Whereas the effect of PPARα ligands on cell and peroxisome proliferation is clearly distinct between rodent and human liver cells, the effect of PPARα ligands on the expression of genes involved in lipid metabolism is generally well conserved between the different species. Indeed, numerous genes connected to lipid metabolism are commonly induced by PPARα ligands in mouse and human hepatocytes, including prototypical PPARα targets such as *CPT1A*, *ACOX1*, *FABP1*, and *HMGCS2* [20]. A recent review on PPARα summarizes the conclusions that can be reached from the use of human liver model systems [19]. In particular, it was concluded that PPARα in human liver is able to effectively induce the expression of genes involved in numerous lipid metabolic pathways. In addition, similar to what is observed in mouse liver, PPARα activation in human liver causes the down-regulation of a large number of genes involved in various immunity-related pathways [19, 21].

The specific model systems used to study PPARα in human liver vary from hepatoma cell lines such as HepG2 to human primary hepatocytes [20, 22–24], human precision cut liver slices [21], and mice expressing human PPARα [25, 26]. Each of these model systems have their specific advantages and disadvantages. An alternative model consist of chimeric mice carrying human liver cells. These mice are generated by transplanting human hepatocytes into albumin enhancer–driven urokinase-type plasminogen activator transgenic/severe combined immunodeficiency (uPA/SCID) mice, leading to replacement of the host hepatocytes at a repopulation rate exceeding 70% [27, 28]. An important advantage of the hepatocyte humanized livers is that the hepatocytes still replicate, in contrast to cultured human primary hepatocytes or liver slices. Recently, we used these PXB mice to study the in vivo effect of PPARα activation using fenofibrate on peroxisome proliferation and the growth of human hepatocytes in mice, leading to the conclusion that rodent data on PPARα-induced hepatocarcinogenesis cannot be accurately extrapolated to humans [2]. Here, we performed transcriptomics analysis on the effect of fenofibrate in chimeric mice with hepatocyte humanized livers and compared the results with other relevant transcriptomics datasets.

## Results

**Fig. 1:**
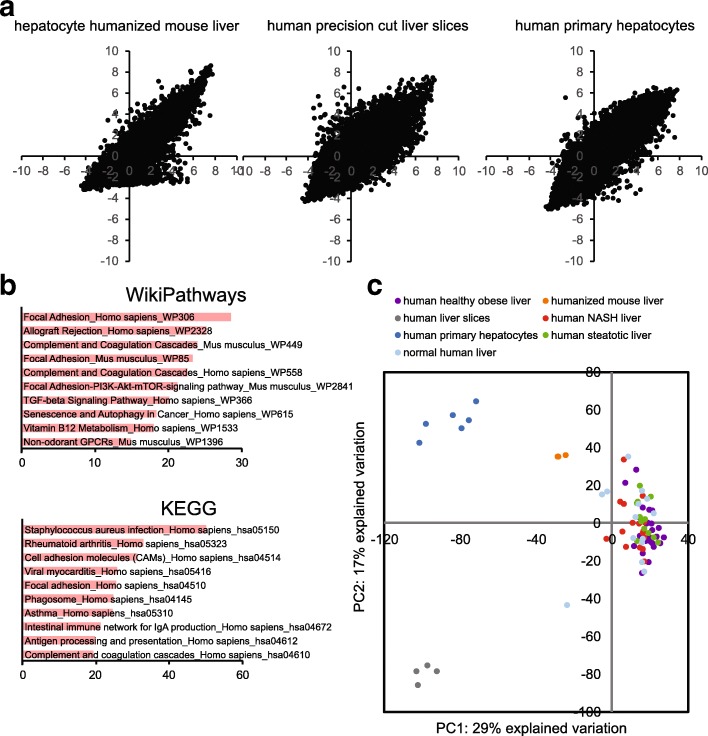
Comparative transcriptomics analysis of different human liver model systems. Transcriptomics was carried out on human liver biopsies (GSE48452) [55], hepatocyte humanized livers, primary human hepatocytes (GSE76148) [24], and human precision-cut liver slices (GSE17251) [21]. **a** Scatter plot analysis of normalized expression values comparing the whole genome expression profile of the human liver biopsies (x-axis) with the whole genome expression profile of the hepatocyte humanized livers (left panel), human precision-cut liver slices (middle panel), and human primary hepatocytes (right panel) (all y-axis). **b** Genes that were expressed at much higher levels in human liver biopsies than in hepatocyte humanized livers (Δ^2^log[normalized mean expression value] > 3) were imported into the Enrichr tool (http://amp.pharm.mssm.edu/Enrichr/index.html) [31, 32]. The 10 pathways with the highest combined score are shown. **c** Principle component analysis was performed to compare the transcriptome of human liver of different types of subjects (normal, healthy obese, steatosis, and NASH) with hepatocyte humanized mouse liver and other liver models, including human primary hepatocytes and human liver slices

First, we compared the whole genome expression profile of human liver biopsies with the whole genome expression profile of hepatocyte humanized mouse livers, human primary hepatocytes, and human precision-cut liver slices. Scatter plot analysis of normalized expression values revealed that a substantial number of genes that showed expression in human liver tissue were minimally (or not) expressed in hepatocyte humanized livers (Fig 1a). Pathway analysis on this differentially expressed set of genes showed overrepresentation of genes involved in focal adhesion, complement and coagulation, and various immune-related pathways (Fig. 1b), which likely reflects the repopulation of the transplanted human hepatocytes in an immuno-deficient host. Interestingly, for highly expressed genes, hepatocyte humanized livers more closely resembled actual human liver tissue as compared to human primary hepatocytes and human precision-cut liver slices, as reflected by the smaller scatter at the high expression range.

Besides the scatter analysis, we utilized principle component analysis to compare the transcriptome of human liver of different types of subjects (normal, healthy obese, steatosis, and NASH) with hepatocyte humanized mouse liver and other liver models, including human primary hepatocytes and human liver slices (Fig. 1c). The results clearly indicate that the gene expression profiles of hepatocyte humanized liver samples are much closer to human liver as compared to human primary hepatocytes and human liver slices. Interestingly, no clear separation between the different groups of human subjects was found, suggesting that the different liver histological phenotypes do not have a distinctive gene expression profile.

**Fig. 2:**
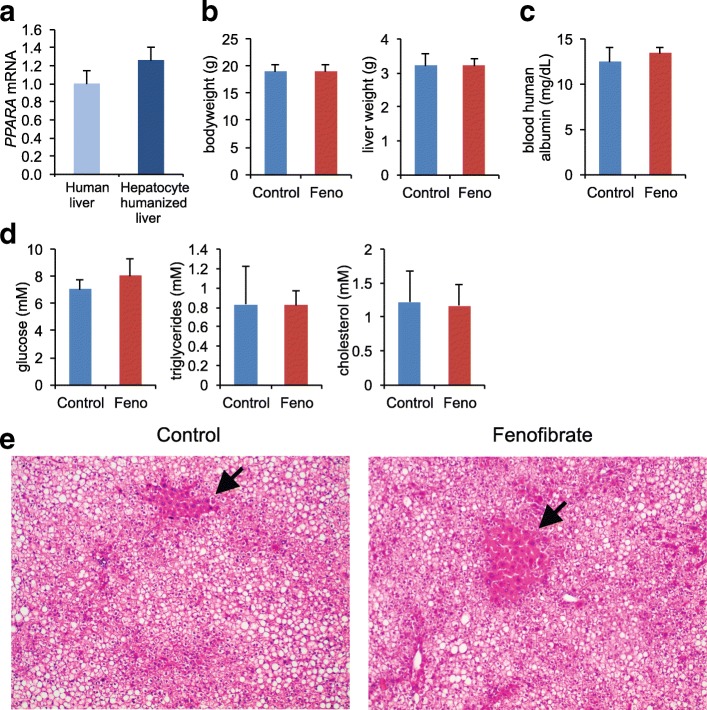
Fenofibrate does not cause any changes in basic parameters in hepatocyte humanized mice. Chimeric mice containing hepatocyte humanized livers were treated with 300 mg/kg fenofibrate daily for 4 days. Control mice received vehicle only. **a** mRNA expression of *PPARα* in 15 human liver biopsies collected during bariatric surgery and in liver samples from 3 chimeric mice containing hepatocyte humanized livers. **b** Bodyweight and liver weight. **c** Blood human albumin concentration. **d** Plasma concentration of glucose, triglycerides and cholesterol. Error bars represent SEM. *N* = 3 per group. **e** Histological examination of livers of chimeric mice containing hepatocyte humanized livers that received control or fenofibrate treatment. Hematoxilin and eosin staining was carried out according to standard protocols. Images are at 200× magnification. A section containing mouse hepatocytes is indicated (arrow). The mouse hepatocytes are present in clusters of non-steatotic cells, with the steatotic human hepatocytes taking up the remainder of the area. In addition, human hepatocytes show a light eosin staining while mouse hepatocytes are highly eosinophilic

As a final validation of the model, mRNA expression levels of PPARα in hepatocyte humanized livers were similar to the levels measured in human liver biopsies (Fig. 2a). These data support the notion that hepatocyte humanized livers are a suitable model for human liver, with some restrictions.

To study the effect of PPARα activation on gene expression in hepatocyte humanized livers, chimeric mice with hepatocyte humanized livers were given fenofibrate or vehicle at a daily oral dose of 300 mg/kg for 4 days. Fenofibrate has a similar affinity for mouse and human PPARα [29]. Fenofibrate did not affect bodyweight or liver weight (Fig. 2b). Also, blood albumin (Fig. 2c), as well as plasma glucose, triglycerides and cholesterol levels were not significantly different between the fenofibrate and control-treated mice (Fig. 2d). Histological examination of the H&E-stained livers showed clearly distinctive clusters of human and mouse hepatocytes. Human hepatocytes showed a light eosin staining while mouse hepatocytes were highly eosinophilic (Fig. 2e). In contrast to the mouse hepatocytes, the human hepatocytes exhibited excessive lipid accumulation (micro- and macrosteatosis), as previously demonstrated [30]. In agreement with our previous study [2], fenofibrate increased the size of the mouse hepatocytes but did not affect the morphology of the human hepatocytes. A tendency toward reduced steatosis by fenofibrate was observed in the sections of the liver populated by human hepatocytes (Fig. 2e).

**Fig. 3:**
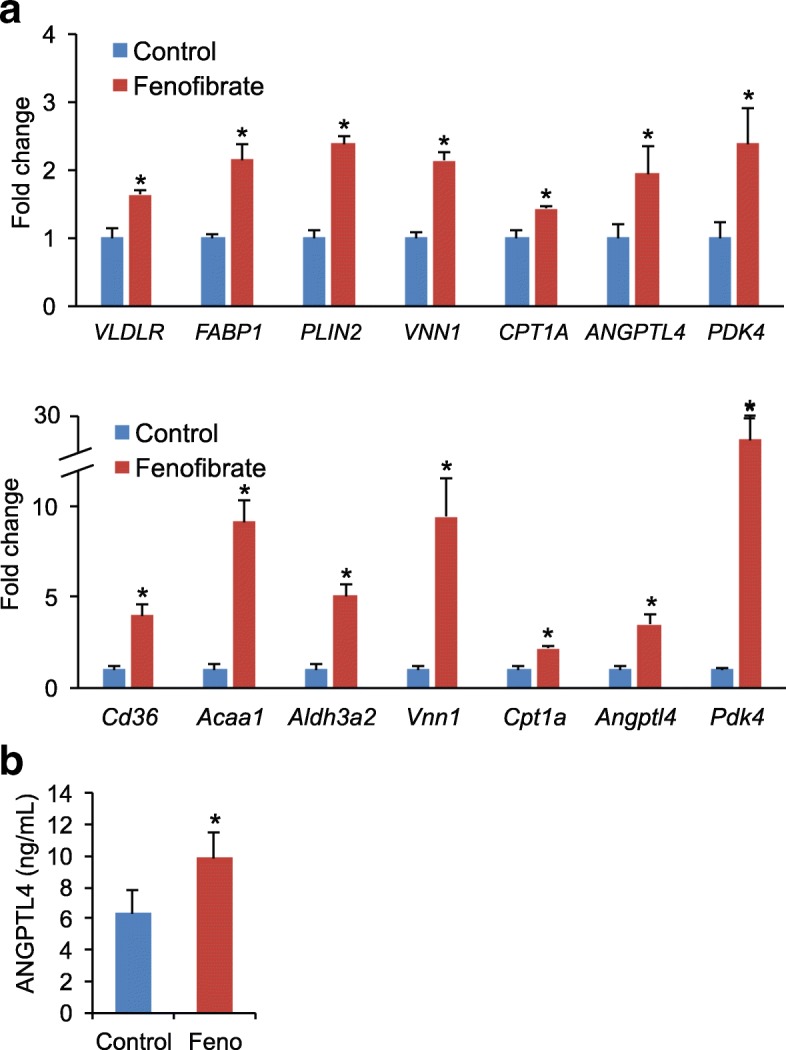
Parallel induction of mouse and human PPARα target genes by fenofibrate in hepatocyte humanized livers. **a** qPCR was performed on cDNA generated from livers of control-treated and fenofibrate-treated mice containing hepatocyte humanized livers, using human primers (upper panel) or mouse primers (lower panel). **b** Concentration of ANGPTL4 in plasma of control-treated and fenofibrate-treated mice containing hepatocyte humanized livers, as determined by ELISA. Error bars represent SEM. *N* = 3 per group. Asterisks indicate statistically significant difference between control and fenofibrate-treated mice according to Student’s t-test with cut-off of *P* < 0.05

To determine whether fenofibrate treatment stimulated PPARα-dependent gene regulation in the mouse and human hepatocytes, we measured the expression of mouse and human PPARα target genes by qPCR in whole liver cDNA using species-specific primers. Fenofibrate treatment significantly increased the expression of known PPARα target genes in human and mouse hepatocytes (Fig. 3a). The overall inductions in gene expression were more pronounced in the mouse hepatocytes than the human hepatocytes. This was also observed for the genes that were measured in both human and mouse hepatocytes, including *Angptl4*, *Vnn1*, *Pdk4* and *Cpt1a* (Fig. 3a). Consistent with the induction of *ANGPTL4* mRNA, fenofibrate treatment also significantly increased levels of human ANGPTL4 in mouse plasma (Fig. 3b).

To study the difference in whole genome expression between the livers of control- and fenofibrate-treated mice, we performed transcriptomics analysis using the Affymetrix Human Gene 1.1 ST Array Plate. This approach allowed us to specifically determine the gene expression changes in the human hepatocytes and avoid potential interference of mouse hepatocytes.

**Fig. 4:**
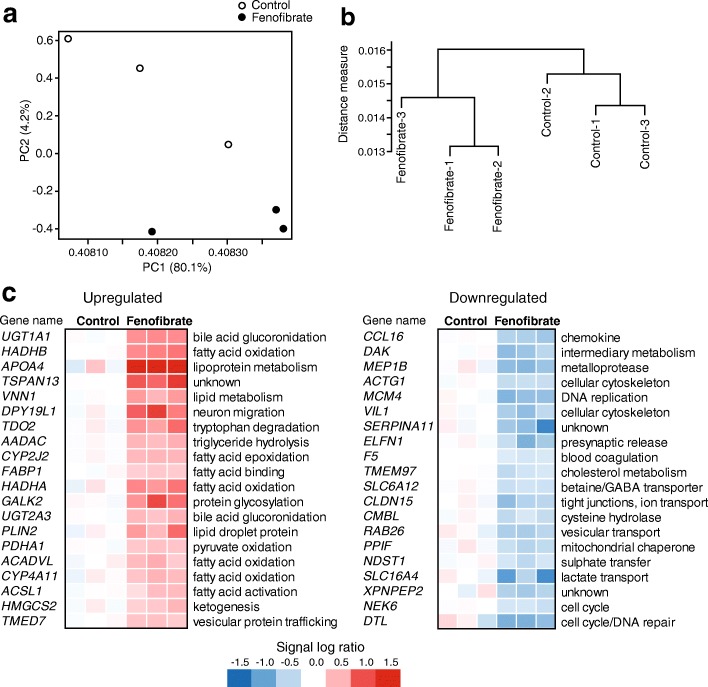
Distinct clustering of livers of control-treated and fenofibrate-treated hepatocyte humanized mice. Transcriptomics was performed on livers of chimeric mice containing hepatocyte humanized livers. Mice were treated with 300 mg/kg fenofibrate daily for 4 days (*n* = 3) or vehicle (control, *n* = 3). **a** Principle component analysis of transcriptomics data from the control- and fenofibrate-treated mice. The graph shows the clear separation of fenofibrate and control groups. **b** Hierarchical clustering of transcriptomics data from the control- and fenofibrate-treated mice. The dendogram reveals the distinct clustering and separation of the fenofibrate and control groups. **c** The top 20 most significantly upregulated and downregulated genes by fenofibrate were ranked according to statistical significance (IBMT *P*-value). The changes in gene expression are expressed relative to the mean of the control group as a signal log ratio

**Fig. 5:**
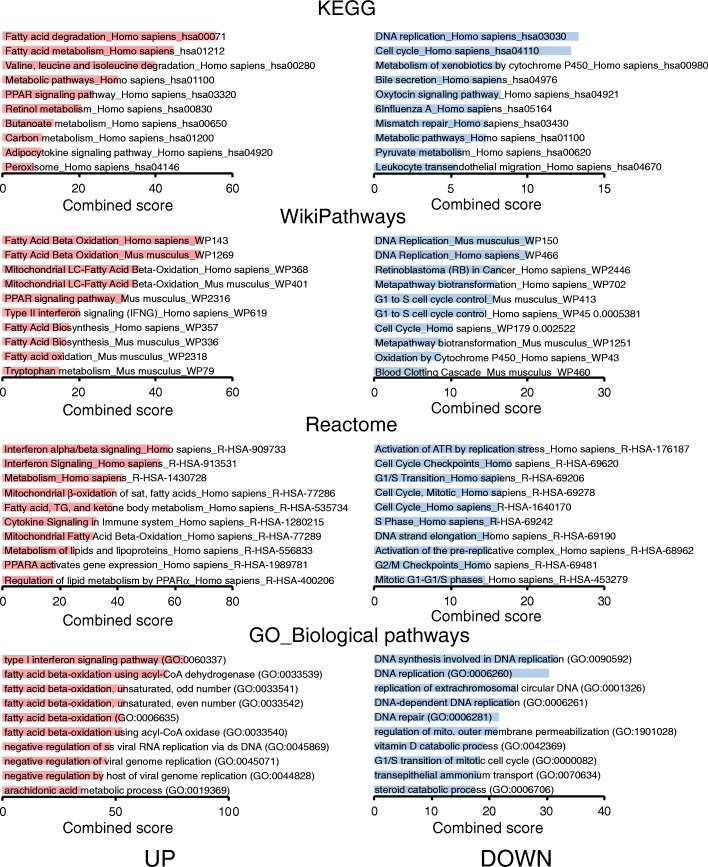
Pathway analysis of differential gene expression between control-treated and fenofibrate-treated hepatocyte humanized mice. Transcriptomics was performed on livers of hepatocyte humanized mice treated with 300 mg/kg fenofibrate daily for 4 days (*n* = 3) or vehicle (control, *n* = 3). Genes that were significantly upregulated (red bars) or downregulated (blue bars) by fenofibrate (IBMT *P*-value< 0.005) were imported into the Enrichr tool (http://amp.pharm.mssm.edu/Enrichr/index.html) [31, 32]. The 10 pathways with the highest combined score are shown, in the following specific categories: KEGG, WikiPathways, Reactome, Gene ontology

Principle component analysis of the transcriptomics data showed that the livers of the fenofibrate-treated hepatocyte humanized mice clearly separated from the livers of the control-treated hepatocyte humanized mice (Fig. 4a). The liver samples from the fenofibrate-treated mice showed less variation than the liver samples from the control-treated mice. A dendogram confirmed the separate clustering of the two sets of samples (Fig. 4b). After filtering, a total of 282 genes was found to be significantly different between fenofibrate- and control-treated mice (*P* < 0.01), of which 159 genes were significantly higher and 123 genes were significantly lower in the fenofibrate-treated mice. The top 20 of most highly induced and repressed genes by fenofibrate is shown in Fig. 4c. The list of induced genes contains many established PPARα targets connected to lipid metabolism, including *FABP1*, *HADHB*, *HADHA*, *VNN1*, *PLIN2*, *ACADVL* and *HMGCS2*. The list of repressed genes is very diverse and does not reveal a common pathway. It includes cytokines (*CCL16*), coagulation factors (*F5*), structural proteins (*ACTG1*), transporters (*SLC16A4/SLC6A12*, and enzymes (*DAK, PPIF*). To gain further insight into the biological pathways induced or repressed by fenofibrate, genes that were significantly upregulated or downregulated by fenofibrate (IBMT *P*-value< 0.005) were further analyzed by Enrichr [31, 32]. The pathways induced by fenofibrate fell into two main categories: fatty acid metabolism and immunity/interferon signaling (Fig. 5). The pathways repressed by fenofibrate were mainly related to cell cycle, mitosis, and DNA synthesis, and to a lesser extent cytochrome P450-mediated biotransformation (Fig. 5).

**Fig. 6:**
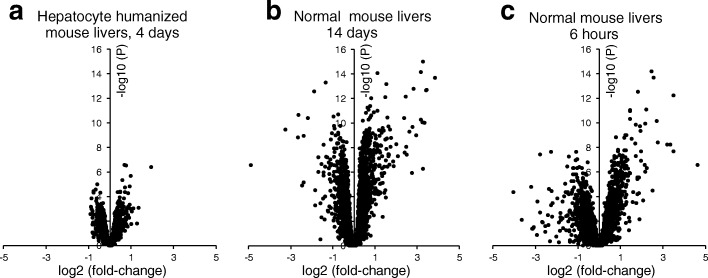
Comparative analysis of the effect of fenofibrate in normal mouse liver and hepatocyte humanized liver. Volcano plots in which ^2^log(fold-change) is plotted against -^10^log(*P*-value) for **a** Treatment of chimeric mice containing hepatocyte humanized livers with 300 mg/kg fenofibrate daily for 4 days as compared to control (*n* = 3 per group); **b** Treatment of wildtype mice with fenofibrate for 14 days via the feed (0.03 wt/wt, equivalent to approximately 1 mg/mouse/day) as compared to control (*n* = 8 per group); **c** Treatment of wildtype mice with fenofibrate for 6 h via a single oral gavage of 4 mg/mouse as compared to control (*n* = 4–5 per group)

Previously, we performed transcriptomics analysis on mouse livers harvested either 6 h after a single oral dose of fenofibrate (4 mg/mouse) or harvested from mice dosed daily with fenofibrate for 14 days by mixing it in the feed (0.03 wt/wt, equivalent to approximately 1 mg/mouse/day). To compare the effect of fenofibrate in normal mouse liver and hepatocyte humanized liver, we performed a comparative analysis of the three transcriptomics datasets. Volcano plot showed that the two week dosing with fenofibrate in normal mice had a much bigger impact on liver gene expression as compared to the fenofibrate treatment in the hepatocyte humanized mice, which is not surprising given the longer duration of the treatment (Fig. 6). Surprisingly, the single treatment of normal mice with 4 mg of fenofibrate also had a more pronounced effect on liver gene expression as compared to the 4-day treatment of the hepatocyte humanized mice with ~6 mg of fenofibrate per day (Fig. 6). These data suggest that in vivo, human liver cells are less sensitive to the effect of fenofibrate as compared to mouse liver cells, confirming the results of the qPCR on the hepatocyte humanized livers. Unfortunately, no transcriptomics dataset was available from normal mice treated with fenofibrate at the same dose and for the same duration as the hepatocyte humanized mice.

**Fig. 7:**
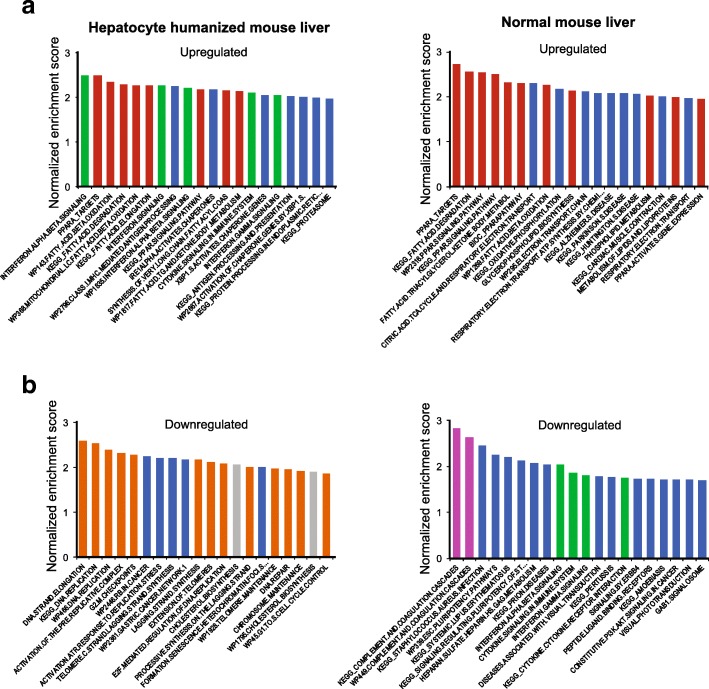
Comparative pathway analysis of the effect of fenofibrate in normal mouse liver and hepatocyte humanized liver. Gene set enrichment analysis was performed on the effect of treatment of chimeric mice containing hepatocyte humanized livers with 300 mg/kg fenofibrate daily for 4 days (*n* = 3 per group), and on the effect of treatment of wildtype mice with fenofibrate for 14 days via the feed (0.03 wt/wt, equivalent to approximately 1 mg/mouse/day, *n* = 8 per group). The top 20 most highly upregulated and downregulated genesets are shown, ranked according to normalized enrichment score. **a** The top 20 most highly upregulated genesets in hepatocyte humanized livers (left panel) and normal mouse livers (right panel). **b** The top 20 most highly downregulated genesets in hepatocyte humanized livers (left panel) and normal mouse livers (right panel). Genesets related to cytokine/interferon signalling are shown in green, genesets related to PPAR signalling and fatty acid oxidation are in red, genesets related to cholesterol synthesis are in grey, genesets related to DNA synthesis in orange, genesets related to complement and coagulation in violet

**Fig. 8:**
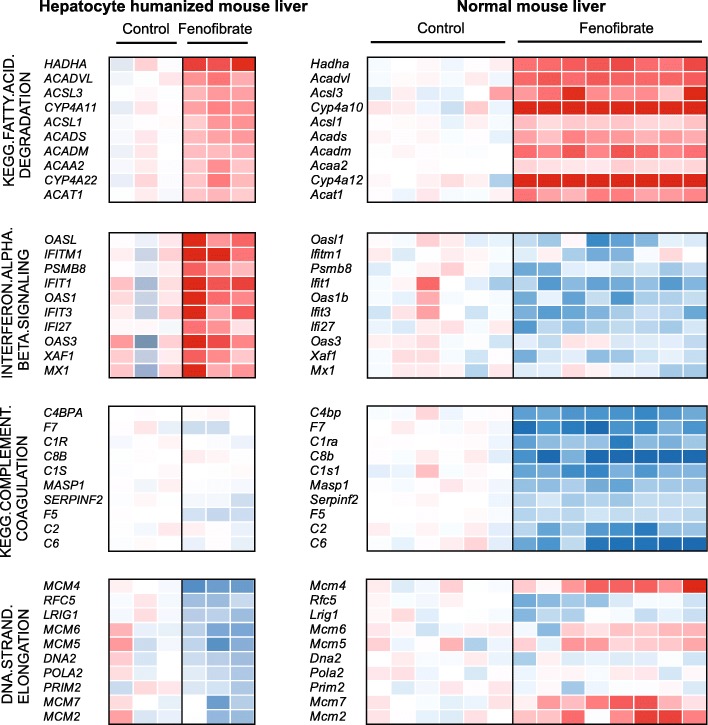
Effect of fenofibrate on specific genesets in normal mouse liver and hepatocyte humanized liver. Gene set enrichment analysis was performed on the effect of treatment of chimeric mice containing hepatocyte humanized livers with 300 mg/kg fenofibrate daily for 4 days (*n* = 3 per group). The 10 most highly ranked genes in the genesets KEGG.FATTY.ACID. DEGRADATION, INTERFERON.ALPHA.BETA.SIGNALING, KEGG.COMPLEMENT. COAGULATION, and DNA.STRAND.ELONGATION are shown. In parallel, expression changes of the same genes are shown in wildtype mice treated with fenofibrate for 14 days via the feed (0.03 wt/wt, equivalent to approximately 1 mg/mouse/day, *n* = 8 per group)

To further compare the effects of fenofibrate between normal mouse liver and hepatocyte humanized liver, we performed gene set enrichment analysis (GSEA). As expected, pathways covering PPARα signaling and fatty acid oxidation featured prominently among the most significantly induced pathways in both normal mouse liver and hepatocyte humanized liver (Fig. 7a, red). The induction by fenofibrate of genes that are part of the geneset KEGG.FATTY.ACID.DEGRADATION is illustrated in Fig. 8, showing a consistent pattern of upregulation in hepatocyte humanized liver and normal mouse liver. Surprisingly, certain immune-related pathways such as interferon signaling were strongly upregulated in hepatocyte humanized liver, but were markedly downregulated in normal mouse liver (Fig. 7a and b, green). The differential regulation of the geneset INTERFERON.ALPHA.BETA.SIGNALING by fenofibrate in hepatocyte humanized liver and normal mouse liver is visualized in Fig. 8.

With respect to down-regulated pathways, it was observed that many of the most significantly downregulated pathways by fenofibrate in hepatocyte humanized liver were related to DNA synthesis (Fig. 7b, orange), which was not observed in normal mouse liver. Indeed, in normal mouse liver, fenofibrate significantly upregulated pathways related to cell cycle and DNA synthesis, although they were not in the top 20 pathways. The differential regulation of the geneset DNA.STRAND.ELONGATION by fenofibrate between hepatocyte humanized liver and normal mouse liver is visualized in Fig. 8. Interestingly, whereas certain genes such as *RFC5* are consistently downregulated by fenofibrate in the two models, other genes such as *MCM4* show opposite regulation by fenofibrate in hepatocyte humanized liver and normal mouse liver (Fig. 8).

The two most significantly repressed pathways by fenofibrate in normal mouse liver were related to complement and coagulation (Fig. 7b, violet). With the exception of F5 and perhaps F7, the suppressive effect of fenofibrate on complement and coagulation factors in normal liver was poorly reproduced in hepatocyte humanized livers (Fig. 8). Finally, pathways related to cholesterol biosynthesis were downregulated by fenofibrate in hepatocyte humanized liver, which also was not seen in normal mouse liver (Fig. 7b, grey). Overall, GSEA shows that the effects of fenofibrate in normal mouse liver and hepatocyte humanized liver are quite distinct, especially in relation to DNA synthesis pathways and interferon signaling pathways.

**Fig. 9:**
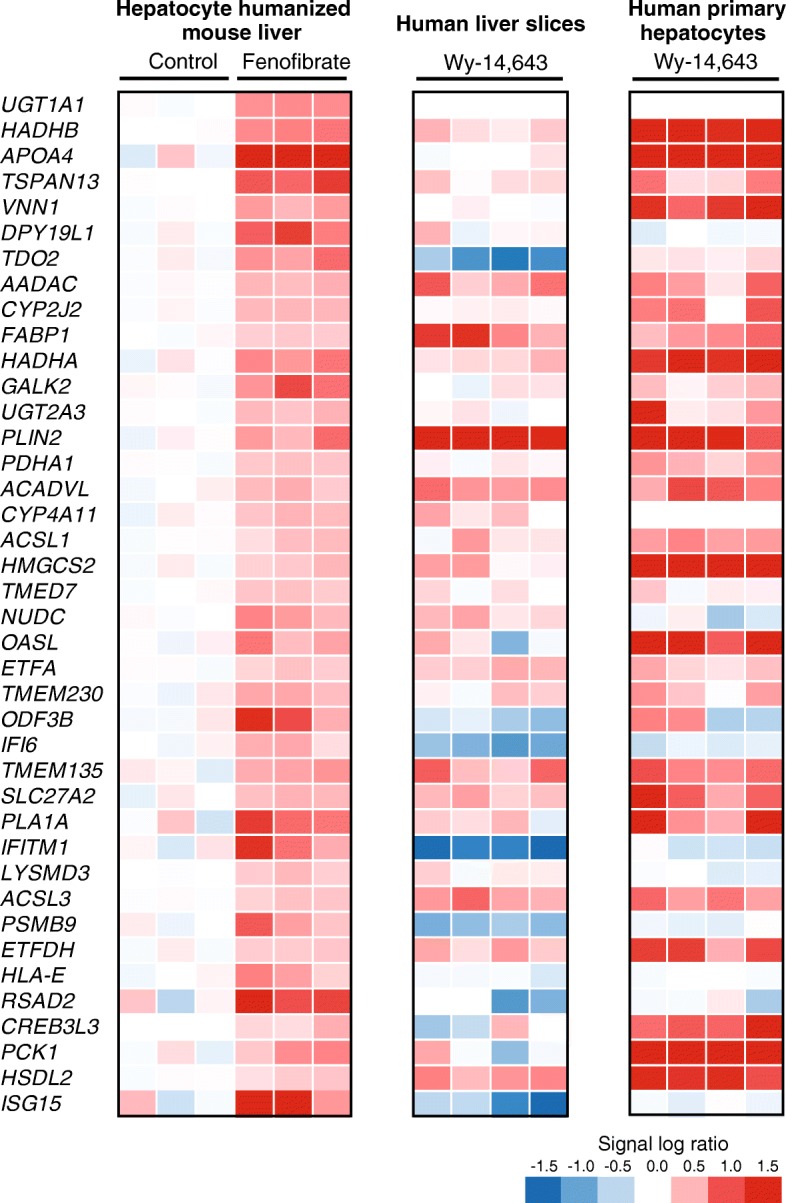
Comparative analysis of PPARα-induced genes between different experiments. Transcriptomics was performed on livers of chimeric mice containing hepatocyte humanized livers. Mice were treated with 300 mg/kg fenofibrate daily for 4 days (*n* = 3) or vehicle (control, *n* = 3). A) The top 40 most significantly induced genes by fenofibrate in hepatocyte humanized livers were ranked according to IBMT P-value. In parallel, the expression profiles of the same genes in two independent microarray datasets are shown. The first dataset is derived from human precision cut liver slices treated with the PPARα agonist Wy-14,643 (100 μM) for 24 h (*n* = 4, GSE17251) [21]. The second dataset is derived from human primary hepatocytes treated with the PPARα agonist GW7647 (1 μM) for 24 h (*n* = 4, GSE53399) [56]

We previously studied the effect of PPARα activation in human primary hepatocytes and human precision cut liver slices. The studies were not carried out with fenofibrate but with Wy14,643, another PPARα agonist, precluding a whole genome comparison with the study in chimeric mice carrying hepatocyte humanized livers. Nevertheless, we took the top 40 most highly induced genes by fenofibrate in hepatocyte humanized livers and compared the fenofibrate-induced expression changes with the Wy-14,643-induced expression changes in human primary hepatocytes and human precision cut liver slices (Fig. 9). The most apparent difference was the regulation of several interferon-sensitive genes, including *IFI6*, *IFITM1*, *PSMB9* and *ISG15*, which were upregulated by fenofibrate in the hepatocyte humanized mouse livers but downregulated by Wy-14,643 in human primary hepatocytes and human precision cut liver slices. Other genes, nearly all representing genes involved in lipid metabolism, were consistently induced by PPARα activation in the three model systems, with in general the highest fold-inductions observed in human primary hepatocytes.

## Discussion

We previously showed that chimeric mice with hepatocyte humanized livers represent an appropriate model to investigate the pharmacological effects of fibrates on human liver [2]. By harbouring clusters of mouse and human hepatocytes, the hepatocyte humanized livers are also an ideal tool to study the parallel effects of a particular treatment on mouse and human hepatocytes. The main findings of the present study are: 1) The hepatocyte humanized livers recapitulate the principal effects of PPARα activation on lipid metabolism revealed by other model systems of human liver. 2) The effects of PPARα activation on gene expression in mice with hepatocyte humanized livers were modest compared to normal mouse liver, which is unlikely due to different treatment protocols. 3) Pathways connected to DNA synthesis were downregulated by fenofibrate in mice with hepatocyte humanized livers, yet are upregulated by fenofibrate in normal mouse livers. 4) Pathways connected to interferon/cytokine signalling were upregulated by fenofibrate in mice with hepatocyte humanized livers, yet are downregulated by fenofibrate in normal mouse liver. 5) Chimeric mice with hepatocyte humanized livers can be used to study the effect of activation of PPARα and other nuclear receptors on secretion of hepatokines into plasma.

**Fig. 10:**
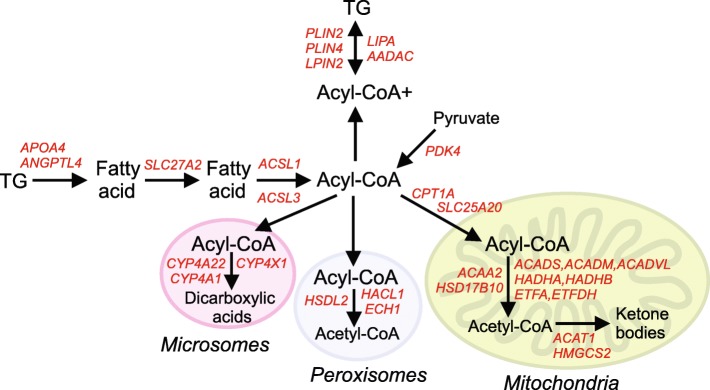
Role of fenofibrate-induced genes in cellular lipid metabolism. Transcriptomics was performed on livers of chimeric mice containing hepatocyte humanized livers. Mice were treated with 300 mg/kg fenofibrate daily for 4 days (*n* = 3) or vehicle (control, *n* = 3). Genes significantly induced by fenofibrate (IBMT *P*-value > 0.02) and with known roles in cellular lipid metabolism were selected. Their roles in specific pathways of cellular lipid metabolism is illustrated

Studies using human liver model systems, including HepG2 cells, human primary hepatocytes, human precision cut liver slices, and PPARα-humanized mice, have yielded detailed information about the effects of PPARα activation on gene regulation in human liver. The results have been summarized in a recent review [19], highlighting the pivotal role of PPARα in governing various metabolic processes and pathways in human liver. Our transcriptomics study in mice with hepatocyte humanized livers confirms the important role of PPARα in regulating lipid metabolism in human liver. Many of the most highly induced genes are well-known targets of PPARα involved in lipid metabolism, including *FABP1*, *ANGPTL4*, *PDK4*, *HADHA*, *HADHA*, *PLIN2*, and *ACADVL*. The position of the fenofibrate-induced genes in cellular lipid metabolism is illustrated in Fig. 10.

Chronic treatment of rodents with peroxisome proliferators causes hepato-carcinogenesis, while short-term treatment promotes hepatocyte and peroxisome proliferation [15–17, 33]. These effects are dependent on induction of several genes involved in DNA synthesis, cell proliferation, and peroxisomal biogenesis and are known to be mediated by PPARα [34]. The pro-carcinogenic effects of peroxisome proliferators led to concerns about their potential hepato-carcinogenicity in humans and triggered numerous studies aimed at investigating the effect of PPARα in human liver model systems. Collectively, these studies have dispelled the notion that peroxisome proliferators are hepato-carcinogenic in humans and have also yielded a wealth of information about the role of PPARα in human liver [19, 35]. In this study, we found that PPARα activation in chimeric mice with hepatocyte humanized livers causes the downregulation of genes and pathways connected to DNA synthesis, further strengthening the notion that the effects of PPARα activation on DNA synthesis, cell proliferation and hepato-carcinogenesis are distinct between mouse liver and human liver. The differential regulation is vividly illustrated by *MCM4*, which was markedly downregulated by fenofibrate in hepatocyte humanized liver but strongly upregulated by fenofibrate in normal mouse liver. Our data thus further mitigate concerns about the hepato-carcinogenic effect of peroxisome proliferators in humans.

One of the major discrepancies between the effect of PPARα activation in hepatocyte humanized liver and normal mouse liver is the regulation of immune-related pathways, especially interferon signaling. Whereas PPARα activation causes the downregulation of interferon signaling in mouse liver, it led to upregulation of interferon signaling in hepatocyte humanized liver. Intriguingly, PPARα activation caused the downregulation of interferon signaling in human precision cut liver slices [21]. Whether the upregulation of interferon signaling by PPARα activation in hepatocyte humanized liver is an artefact of the interaction between human hepatocytes and mouse Kupffer cells, is related to the immune-deficiency in the SCID host mice, or in fact most accurately reflects the response to PPARα activation in human liver remains to be established.

Another pathway that appears to be differentially regulated by PPARα in hepatocyte humanized liver and normal mouse liver is complement and coagulation. The suppression of this pathway by fenofibrate and other PPARα activators in normal mouse liver confirms previous analyses [36, 37], and was suggested to be an energy-saving mechanism [38]. With the exception of *F5* and possibly *F7*, we did not observe any downregulation of coagulation genes by fenofibrate in hepatocyte humanized liver. With respect to the complement pathway, previously we demonstrated that mannose-binding lectin (MBL2)—the primary component of the lectin branch of the complement system—is a target of PPARα and is markedly induced by PPARα activation in primary hepatocytes and human liver slices [21, 39]. However, mRNA levels of *MBL2* and other complement-related genes were not altered by fenofibrate in hepatocyte humanized liver. In mouse liver, *Mbl2* and other complement-related genes such as *C8b* and *C9* are downregulated by PPARα activation (Fig. 8) [36, 39]. These data indicate that complement-related genes are differentially regulated by PPARα in various model systems.

Activation of PPARα alters the expression of a number of apolipoproteins, which may account for the plasma triglyceride-lowering and HDL-raising effects of PPARα agonists in human patients [40]. For instance, fenofibrate was found to induce *APOA1* expression in human primary hepatocytes and elevate plasma APOA1 levels in humans subjects [41, 42]. Similarly, *APOA5* was identified as a direct PPARα target and was shown to be induced by PPARα activators in human and cynomolgus hepatocytes [43, 44]. Consistent with these findings, administration of the PPARα agonist LY570977 to cynomolgus monkeys increased serum APOA5 concentrations by 2-fold [45]. PPARα activation has also been shown to regulate APOC3. Specifically, fenofibrate lowered *APOC3* mRNA in human primary hepatocytes, concomitant with reduced secretion of APOC3 in the culture medium [46]. Interestingly, in hepatocyte humanized mouse liver, fenofibrate treatment did not significantly change the expression of *APOA1* and *APOC3* mRNA, while it increased mRNA levels of *APOA4* (fold-change = 3.9, *P* < 0.0001), and *APOA5* (fold-change is 1.4, *P* < 0.05). Our data, together with data from other human liver model systems, question the regulation of *APOA1* and *APOC3* mRNA by PPARα activation in human liver.

The residual presence of mouse hepatocytes in the hepatocyte humanized mouse livers allowed for direct comparison of the effect of fenofibrate on human and mouse hepatocytes. The results show that the fold-changes in expression of the same genes are more pronounced in mouse as compared to human hepatocytes, verifying the notion that human hepatocytes are less sensitive to PPARα activation.

Overall, it is evident that quantitative and qualitative differences in PPARα-mediated gene regulation exist between mouse and human hepatocytes. Unfortunately, this study provides minimal clues as to why the response to PPARα activation is different in mouse and human hepatocytes. The lower fold-induction of genes related to lipid metabolism by fenofibrate in human versus mouse hepatocytes cannot be attributed to a lower expression of PPARα. Also, the relative binding affinity of fenofibrate for mPPARα and hPPARα appears to be similar [29]. Studies with humanized PPARα mice suggest that differences in intrinsic properties of the mouse and human PPARα protein are responsible for the qualitative differences in gene regulation between the two proteins. Indeed, mice expressing human PPARα do not show hepatomegaly and induction of cell cycle control genes upon PPARα activation [47]. By contrast, quantitative differences in gene regulation by PPARα between human and mouse hepatocytes may be attributed to differences in the epigenetic landscape.

The use of chimeric mice with hepatocyte humanized livers in principle allows for study of the secretion of human liver proteins into the blood. Indeed, we were able to detect human ANGPTL4 in blood plasma of mice with hepatocyte humanized livers. Furthermore, following the induction of *ANGPTL4* mRNA by PPARα, treatment of the mice with fenofibrate significantly increased plasma ANGPTL4 levels. These data are consistent with the increase in plasma ANGPTL4 levels in subjects treated with fenofibrate [39, 48]. Intriguingly, the absolute level of ANGPTL4 in plasma of mice with hepatocyte humanized livers was similar to the levels observed in human subjects, suggesting that liver is the primary source of ANGPTL4 in plasma. Overall, these data suggest that chimeric mice with hepatocyte humanized livers are a suitable model to study the secretion of human liver proteins into the blood.

In our study, the livers of the hepatocyte humanized mice were very fatty, which was observed specifically in the liver sections populated by human hepatocytes. It has been demonstrated that the elevated lipid storage is likely due to a deficiency of the human growth hormone [30]. Whether the difference in lipid storage between mouse and human hepatocytes is in any way connected to differences in PPARα expression or function remains unclear. The excess lipid storage in the hepatocyte humanized livers is a potential limitation for the study of lipid metabolism.

## Conclusions

In conclusion, using transcriptomics, we show that chimeric mice containing hepatocyte humanized livers are a highly valuable tool to study the in vivo function of PPARα in human liver. The results confirm the major role of PPARα in the regulation of hepatic lipid metabolism, yet also demonstrate the more modest effect of PPARα activation on target gene induction in human hepatocytes as compared to mouse hepatocytes. The data suggest that PPARα may have a suppressive effect on DNA synthesis in human liver, and a stimulatory effect on interferon/cytokine signalling.

## Methods

### Animals

The animal study was carried out in PXB mouse (Genotype: cDNA-uPA+/wt/SCID, uPA+/wt; B6;129SvEv-Plau, SCID; C.B-17/Icr-scid/scid Jcl) at PhoenixBio Co. Ltd. The mice were acquired from PhoenixBio Co. Ltd. A full description of the generation of these mice can be found elsewhere [49]. Briefly, cryopreserved human hepatocytes from a 2-year-old Hispanic girl were purchased from BD Biosciences (Woburn, MA, USA). After thawing, the hepatocytes were transplanted into hemizygous 2- to 4-week-old cDNA-uPA/SCID mice via the spleen under anesthesia. Six male mice between 12 and 18 weeks of age were used for the study.

Fenofibrate was dissolved in 0.5% hydroxypropyl methylcellulose (Shinestu Kagaku Kogyo, Japan) and administered orally to the mice at a dose of 300 mg/kg once per day for 4 days with a disposable plastic sonde (Fuchigami Kikai Co., Kyoto, Japan). Three mice received fenofibrate and three mice received the control treatment with vehicle only.

The experiment was terminated 24 h after the final dosing with fenofibrate. Mice were anesthetized using isoflurane and a minimum of 300 μL of blood was collected via cardiac puncture into sodium heparinized syringes. The mice were euthanized by exsanguination. Blood was used for measurement of human albumin or centrifuged to obtain plasma. Plasma was subsequently frozen at − 80 °C. Liver tissue was collected and either frozen at − 80 °C or fixed in formaldehyde and further processed for histology. All experimental procedures were conducted in accordance with the guidelines provided by Proper Conduct of Animal Experiments (June 1, 2006; Science Council of Japan) and approved by the Animal Care and use Committee of PhoenixBio Co., Ltd. The animal handling guidelines that were followed in this study were based on the Act on Welfare and Management of Animals (Act No. 105 of October 1, 1973; hereinafter, the Act), The Standards and Norms for the Breeding and Housing of Laboratory Animals (Prime Minister’s Notice #6 of 1980; hereinafter, the Notice) and the Guidelines for Proper Conduct of Animal Experiments (June 1, 2006; Science Council of Japan).

### Plasma measurements

Plasma concentrations of glucose (Sopachem, Ochten, the Netherlands), triglycerides, and cholesterol (Instruchemie, Delfzijl, the Netherlands) were determined following the manufacturers’ instructions. The plasma concentration of ANGPTL4 was determined as previously described [48].

### RNA isolation and qPCR

Total RNA of human and mouse tissue was isolated using TRIzol reagent (Invitrogen). RNA was reverse transcribed using the iScript cDNA Synthesis Kit (Bio-Rad Laboratories BV, Veenendaal, The Netherlands). Messenger RNA levels of selected genes were determined by reverse transcription quantitative PCR using SensiMix (Bioline; GC Biotech, Alphen aan den Rijn, The Netherlands) on a CFX384 real-time PCR detection system (Bio-Rad Laboratories, Veenendaal, the Netherlands). The housekeeping gene 36b4 was used for normalization. Sequences of the primers used are listed in Table 1. Mouse primers and human primers are specific for mouse and human, respectively. Primer pairs contain at least 4 mismatches with the other organism. Primer specificity was assessed using NCBI primer-BLAST.

**Table 1:** Sequences of primers used in qPCR analysis

Name	Forward	Reverse
*36B4/36b4*	CGGGAAGGCTGTGGTGCTG	GTGAACACAAAGCCCACATTCC
*VNN1*	AGTGGCATCTATGCACCCAAT	GGAATCCAGTTGCGAGAGGA
*PPARA*	CAGAACAAGGAGGCGGAGGTC	TTCAGGTCCAAGTTTGCGAAGC
*ANGPTL4*	CGTACCCTTCTCCACTTGGG	GCTCTTGGCGCAGTTCTTG
*CPT2*	GCATACGGGCAGATAAACCACAA	ACAGCATACCCAACACCAAAGC
*PDK4*	TGGAGCATTTCTCGCGCTAC	ACAGGCAATTCTTGTCGCAAA
*VLDLR*	GGTGAAAATGATTGTGACAGTGG	GTGAACTCGTCGGGACTACA
*PLIN2*	ATGGCAGAGAACGGTGTGAAG	CAACTGCAATTTGCGGCTC
*FABP1*	ATGAGTTTCTCCGGCAAGTACC	CTCTTCCGGCAGACCGATTG
*Cd36*	GAGCAACTGGTGGATGGTTT	GCAGAATCAAGGGAGAGCAC
*Acaa1*	AAGGCAGGTTGTCACGCTACT	CCTCAGTTCCCAGGGTATTCAAAG
*Aldh3a2*	CCTGAGCAAAAGTGAACTCAATG	GCTCCAATAATCAGTACGACTCC
*Ppara*	TATTCGGCTGAAGCTGGTGTAC	CTGGCATTTGTTCCGGTTCT
*Angptl4*	GTTTGCAGACTCAGCTCAAGG	CCAAGAGGTCTATCTGGCTCTG
*Plin2*	CTTGTGTCCTCCGCTTATGTC	GCAGAGGTCACGGTCTTCAC
*Pdk4*	TCTACAAACTCTGACAGGGCTTT	CCGCTTAGTGAACACTCCTTC
*Vnn1*	CTTTCCTCGCGGCTGTTTAC	CCTCCAGGTATGGGTAGATCGT

### Microarray analysis

For microarray hybridization, the isolated RNA was further purified using RNeasy Minikit columns (Qiagen). RNA concentrations were measured on a nanodrop ND-1000 UV-Vis spectrophotometer (Isogen, Maarssen, The Netherlands) and analyzed on an Agilent 2100 bioanalyzer (Agilent Technologies, Amsterdam, The Netherlands) with 6000 Nano Chips, according to the manufacturer’s protocol. RNA was judged suitable for array hybridization only if samples exhibited intact bands corresponding to the 18S and 28S ribosomal RNA subunits, and displayed no chromosomal peaks or RNA degradation products.

Purified RNA (100 ng) was labeled with the Ambion WT expression kit (Invitrogen) and hybridized to Affymetrix Human Gene 1.1 ST arrays, provided in plate format (Affymetrix, Santa Clara, CA). Hybridization, washing and scanning of the array plates was performed on an Affymetrix GeneTitan instrument, according to the manufacturer’s recommendations. Normalized expression estimates were obtained from the raw intensity values applying the robust multi-array analysis preprocessing algorithm available in the Bioconductor library AffyPLM with default settings [50, 51]. Probe sets were defined according to Dai et al. [52]. In this method probes are assigned to Entrez IDs as a unique gene identifier. In this study, probes were reorganized based on the Entrez Gene database, build 37, version 1 (remapped CDF v22), which excludes probes from analysis when they have more than 1 mismatch with the human genome, thereby largely assuring the human specificity of the analysis. The entire geneset was condensed by applying an Inter Quartile Range filter of 0.25 and by excluding genes with mean expression level below 20. The *P* values were calculated using an Intensity-Based Moderated T-statistic (IBMT) [53]. Genes were defined as significantly changed when *P* < 0.01. The microarray data were deposited at Gene Expression Omnibus (accession number GSE107041) and can be accessed via: https://www.ncbi.nlm.nih.gov/geo/query/acc.cgi?acc=GSE107041.

Gene set enrichment analysis (GSEA) was used to identify genesets that were enriched among the upregulated or downregulated genes [54]. Genes were ranked based on the IBMT-statistic and subsequently analyzed for over- or underrepresentation in predefined genesets derived from Gene Ontology, KEGG, National Cancer Institute, PFAM, Biocarta, Reactome and WikiPathways pathway databases. Only genesets consisting of more than 15 and fewer than 500 genes were taken into account. Statistical significance of GSEA results was determined using 1000 permutations.

### Statistical analysis

Data are presented as mean ± SEM. Differences between the fenofibrate and control groups were analysed using two-tailed Student’s t-test. *P* < 0.05 was considered as statistically significant.

## Abbreviations

GSEA: Gene Set Enrichment Analysis
PPAR: Peroxisome Proliferator Activated Receptor
